# Providing researchers with online access to NHLBI biospecimen collections: The results of the first six years of the NHLBI BioLINCC program

**DOI:** 10.1371/journal.pone.0178141

**Published:** 2017-06-14

**Authors:** Carol A. Giffen, Elizabeth L. Wagner, John T. Adams, Denise M. Hitchcock, Lisbeth A. Welniak, Sean P. Brennan, Leslie E. Carroll

**Affiliations:** 1 Information Management Services, Inc., Calverton, Maryland, United States of America; 2 Translational Blood Science and Resources Branch, Division of Blood Diseases and Resources, National Heart, Lung, and Blood Institute, Bethesda, Maryland, United States of America; Stellenbosch University Faculty of Medicine and Health Sciences, SOUTH AFRICA

## Abstract

The National Heart, Lung, and Blood Institute (NHLBI), within the United States’ National Institutes of Health (NIH), established the Biologic Specimen and Data Repository Information Coordinating Center (BioLINCC) in 2008 to develop the infrastructure needed to link the contents of the NHLBI Biorepository and the NHLBI Data Repository, and to promote the utilization of these scientific resources by the broader research community. Program utilization metrics were developed to measure the impact of BioLINCC on Biorepository access by researchers, including visibility, program efficiency, user characteristics, scientific impact, and research types. Input data elements were defined and are continually populated as requests move through the process of initiation through fulfillment and publication. This paper reviews the elements of the tracking metrics which were developed for BioLINCC and reports the results for the first six on-line years of the program.

## Introduction

The National Heart, Lung, and Blood Institute (NHLBI) provides global leadership in the prevention and treatment of heart, lung, and blood diseases and supports basic, translational and clinical research in these areas. In 2007 the NHLBI established a Strategic Plan structured around three goals: Goal 1: Form to function; Goal 2: Function to cause; and Goal 3: Cause to cures. Two strategies to accomplish these goals are “t*o develop and facilitate access to scientific research resources”* and “*increase the return from NHLBI population-based and outcomes research”* [1]. In accordance with the 2007 goal-based strategies, the NHLBI established the Biologic Specimen and Data Repositories Information Coordinating Center (BioLINCC) in 2008 to expand the utilization of two unique research resources developed and maintained by the NHLBI. These resources are the NHLBI Biologic Specimen Repository (Biorepository), which has been managed by the Division of Blood Diseases and Resources since 1975, and the NHLBI Data Repository, which has been managed by the Division of Cardiovascular Sciences since 2000.

The primary objective of the BioLINCC program is to maximize the scientific value of historical and contemporary NHLBI biospecimen and data collections by facilitating access by qualified researchers to these research resources, and to enhance utilization by promoting awareness of these resources to the research community. The approach and methods used to establish the program have been described previously [2]. Briefly, the biospecimens in the Biorepository were linked with their study data. A program website (https://biolincc.nhlbi.nih.gov/) was established to enable 1) a public-facing information resource for researchers to learn about the available studies and research resources, 2) private communication workspaces for the online request of these resources, and 3) the supporting infrastructure to facilitate an online Institute review and approval process. Having biospecimens linked with their clinical data allows BioLINCC to conduct detailed searches to identify suitable biospecimens for the proposed research project by specific clinical and phenotypic characteristics of the research subjects as well as by specific biospecimen types, volumes and draw times (e.g., study visits). From an overall Biorepository inventory control perspective, the established linkages along with the detailed biospecimen inventory systems enable BioLINCC to provide comprehensive information to the Institute regarding the impact of fulfilling each request on post-fulfillment Biorepository stock.

At program initiation a comprehensive set of metrics was developed to provide data on how well the program was improving access to the Biorepository. The metrics included data on the efficiency of the workflows used to search, review and distribute biospecimens, as well as characteristics of biospecimen resource users and the scientific impact of their research. This paper describes the rationale and methods used for these metrics, and reports the results obtained for the first six years of online access. We also discuss how the metrics have been used to improve program efficiency and to assist in developing strategies to promote scientific use.

## Methods

### Program visibility–Website hits and registered users

Because the primary mechanism for interface with its researchers is via the BioLINCC website, one measure of program visibility over time is to examine visibility as measured by website access metrics. BioLINCC uses website monitoring software [AWStats 7.2 (build 1.992)] to track the numbers of new and unique users (excluding robots, spiders, worms, etc.). This software also provides a wealth of information on pages visited, downloads, referral sites, and other data which can be used to explore site activity after program promotional events.

Also of interest is the number of unique users who not only view the BioLINCC website but who also become registered users. The database within the website provides information on counts of active registered users over time.

### Biospecimen request parameters and request fulfillment metrics

Numerous data are collected and tracked, starting from the original request submission through termination or fulfillment; for fulfilled requests, data collection continues until BioLINCC receives notification from the user that the research has been completed. Information about the proposed research project which is collected from the user during the request submission includes:

- Numbers, types, and minimum/optimal volumes or quantities (e.g., for DNA) of specimens requested, and the required study time points for specimen draws
- Characteristics of the target population to be searched; for example, affected/unaffected status, treatment arm, study time points
- Summary of the research aims, assays/platforms to be used and an Institutional Review Board (IRB)-approved study protocol
- Whether funding is currently available or is being applied for, and information on the funding source including the NIH or other type of grant number/funding opportunity identifier (if applicable)

To explore the research purposes of fulfilled biospecimen requests, NHLBI program staff provides scientific guidance in the classification of research aims into one of the following four Research Type groups, defined below:

- **Pilot study–**a small scale preliminary study conducted in order to evaluate feasibility, appropriateness of the available biospecimens, and/or effect size prior to the conduct of the larger scale study.
- **Assay validation–**the research aim is to provide documented evidence that an assay does what it is intended to do.
- **Hypothesis generating**–the research aim is to provide documented evidence that can be used to develop a specific, testable prediction.
- **Hypothesis testing**–the research aim is to test a specific prediction based on prior results. Research aims to confirm or refute hypotheses arising from hypothesis generating studies are included in this category.

The study website tracks key dates during the entire process, which are flagged in the system by BioLINCC staff at each key post-submission milestone. These flagged dates are used to compute intervals for use in metrics tables and graphs. It is from these data that simple, overall point-to-point intervals can be computed (e.g., time from submission to fulfillment or termination without fulfillment, time from Institute approval to shipment, etc.). A somewhat more complicated set of time metrics reflects the fact that during the overall request process, responsibility for the next action item often shifts between the different parties (BioLINCC, the Biorepository, the user, the funding group and the NHLBI). A single time metric does not provide the nuances needed to understand where requests may become stalled, and more importantly to identify if actions can be taken to overcome certain obstacles. The flags that are set by BioLINCC are used to sum, in aggregate, the amount of time spent by each party throughout the process.

Additional flags set by BioLINCC staff provide metrics classification information regarding requests which are ultimately closed without fulfillment. These include Request Not Funded, Materials Not Available, Request Requirements (usually related to IRB approval), etc.

Finally, data are gathered to track the scientific impact of the research performed using the requested biospecimens through publications. This is done via requests for annual progress reporting from biospecimen recipients, as well as by literature searches.

### Requestor metrics

An objective of the BioLINCC program is to enhance utilization of stored resources among the wider scientific community, with a particular interest in encouraging their use among early career investigators and among investigators who were not part of the original research. Therefore, certain information related to the requestor is collected during the request process. This enables the tabulation of data related to the effectiveness of the BioLINCC program in reaching out to junior- and mid-tenure researchers, and also to document whether the use of research specimens from archived collections is expanding beyond the investigators who collaborated in the original study. Prior to the establishment of BioLINCC, most requests for biospecimens were either for specimens in the Proprietary Phase, and still under the control of the Parent Study, or were made by investigators who had participated in the Parent Study research.

### Biospecimen utilization metrics

In order to assess utilization of the various biospecimen resources by the research community, the overall percentage of distribution of biospecimens relative to the size of each stored biospecimen collection are calculated. These percentages are then compared to a target of 5% distribution over 5 years. Collections which do not meet this target are periodically evaluated for future reductions and/or enhanced promotional activities.

## Results

### Program visibility–Website hits and registered users

**Fig 1:**
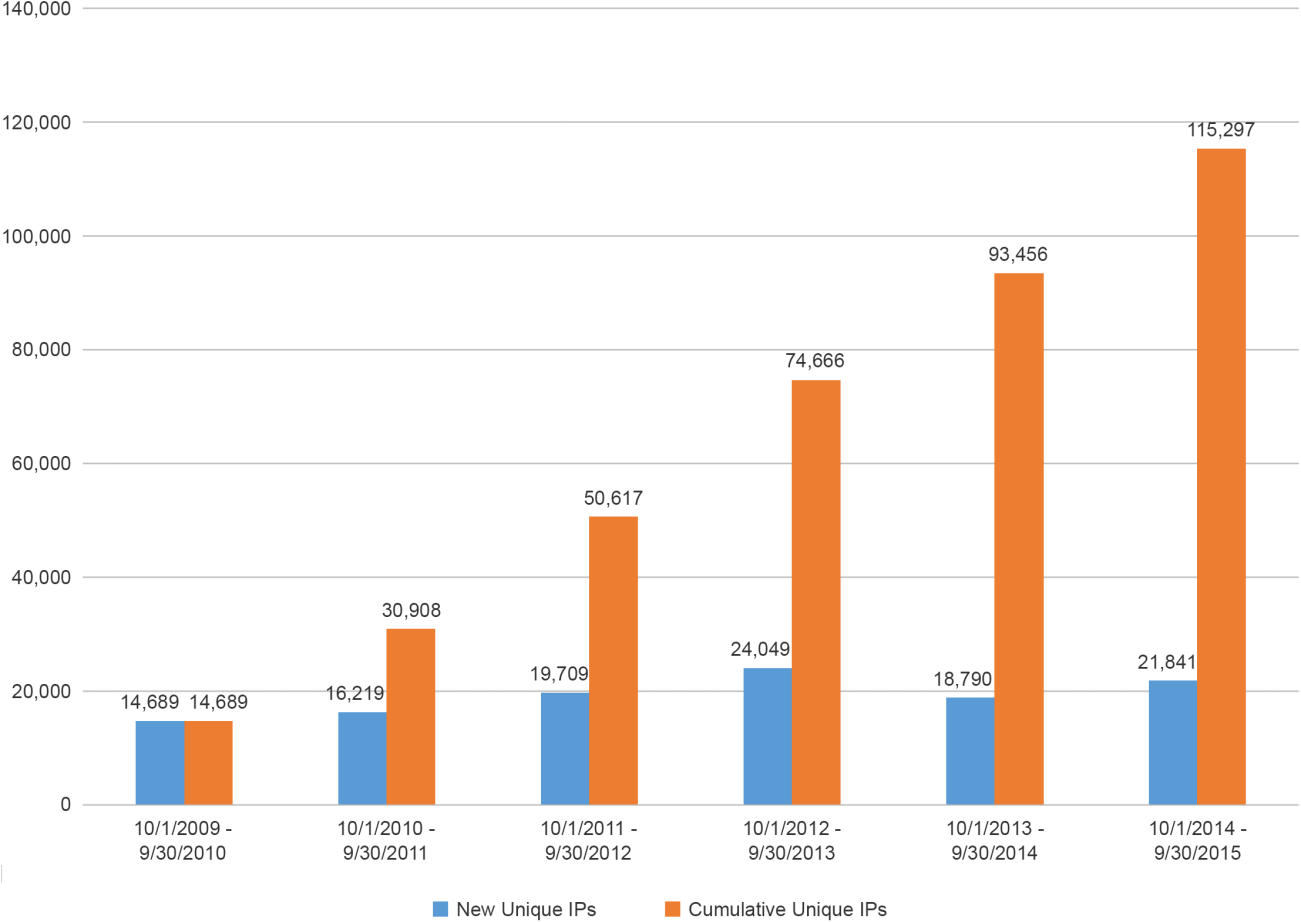
Annual and cumulative access to the BioLINCC website by program year for the 1^st^ six years of operation.

Fig 1 illustrates the annual and cumulative access to the website by program year for the first six full years of operation. This display includes counts of new and unique Internet Protocol (IP) addresses which have accessed the website, and excludes automated access to the site. By the end of the first online year, 14,689 new IP’s had accessed the site; by the end of the sixth online year, 115,297 new and unique users had done so, a 6.8-fold increase.

Over the same timeframe, active registered users increased each year. Although registration is not required to access most of the website content, it is required to submit requests for resources and to receive email notifications of new and updated resources. As such, registration indicates an increased level of interest in the program beyond casual browsing. By the end of its first online year, BioLINCC had 479 active registered users, and by the end of the sixth online year the cumulative active registered user base was 3,938, a 7.2-fold increase.

### Request metrics

Table 1 provides an annual breakdown of biospecimen requests received by website year, and indicates whether requests were fulfilled or ultimately unsuccessful. For fulfilled requests, this table also provides information on the types of research for which biospecimens were supplied. Through the end of the reporting period, a total of 214 biospecimen requests were submitted. Peak years for requests were during the second and third online years because NHLBI grant funding opportunities were made available for research using biospecimens obtained via the BioLINCC program. Two thirds (68%) of the successful requests were intended for hypothesis testing.

**Table 1:** Biospecimen requests and disposition, by website year, and research type for fulfilled requests.

All Requests	Total	Request Disposition		Research Type (Fulfilled Requests Only)			
Website Year		Fulfilled	Unsuccessful	Assay Validation	Hypothesis Testing	Hypothesis Generating	Pilot Study
**10/1/2009–9/30/2010**	27	14	13	2	10	0	2
**10/1/2010–9/30/2011**	57	29	28	2	23	4	0
**10/1/2011–9/30/2012**	69	23	46	7	9	7	0
**10/1/2012–9/30/2013**	24	12	12	1	6	5	0
**10/1/2013–9/30/2014**	14	8	6	1	6	1	0
**10/1/2014–9/30/2015**	23	13	10	0	13	0	0
**Total**	214	99	115	13	67	17	2

The reason for failure for unsuccessful biospecimen requests by online year is provided in Table 2. The single most common reason for the termination of requests for biospecimens has been the lack of investigator funding to perform their proposed research (n = 44, 38%).

**Table 2:** Reasons biospecimen requests were not fulfilled, by website year.

	Unsuccessful Requests—Reason not Fulfilled						
Website Year	Inactivity	Denied	Materials Not Available	Request Requirements	Request Not Funded	Cancelled by User/NOS	Total
10/1/2009–9/30/2010	4	0	7	1	0	1	13
10/1/2010–9/30/2011	1	1	5	2	16	3	28
10/1/2011–9/30/2012	6	2	8	4	23	3	46
10/1/2012–9/30/2013	6	1	3	0	1	1	12
10/1/2013–9/30/2014	3	0	2	0	1	0	6
10/1/2014–9/30/2015	4	0	1	1	3	1	10
Total	24	4	26	8	44	9	115

The overall time to fulfillment is defined as the days from initial request submission through the shipment of biospecimens to the requestor. In the first year the overall median time to fulfillment was 166 days, and by the sixth year that duration had decreased to 117 days.

**Fig 2:**
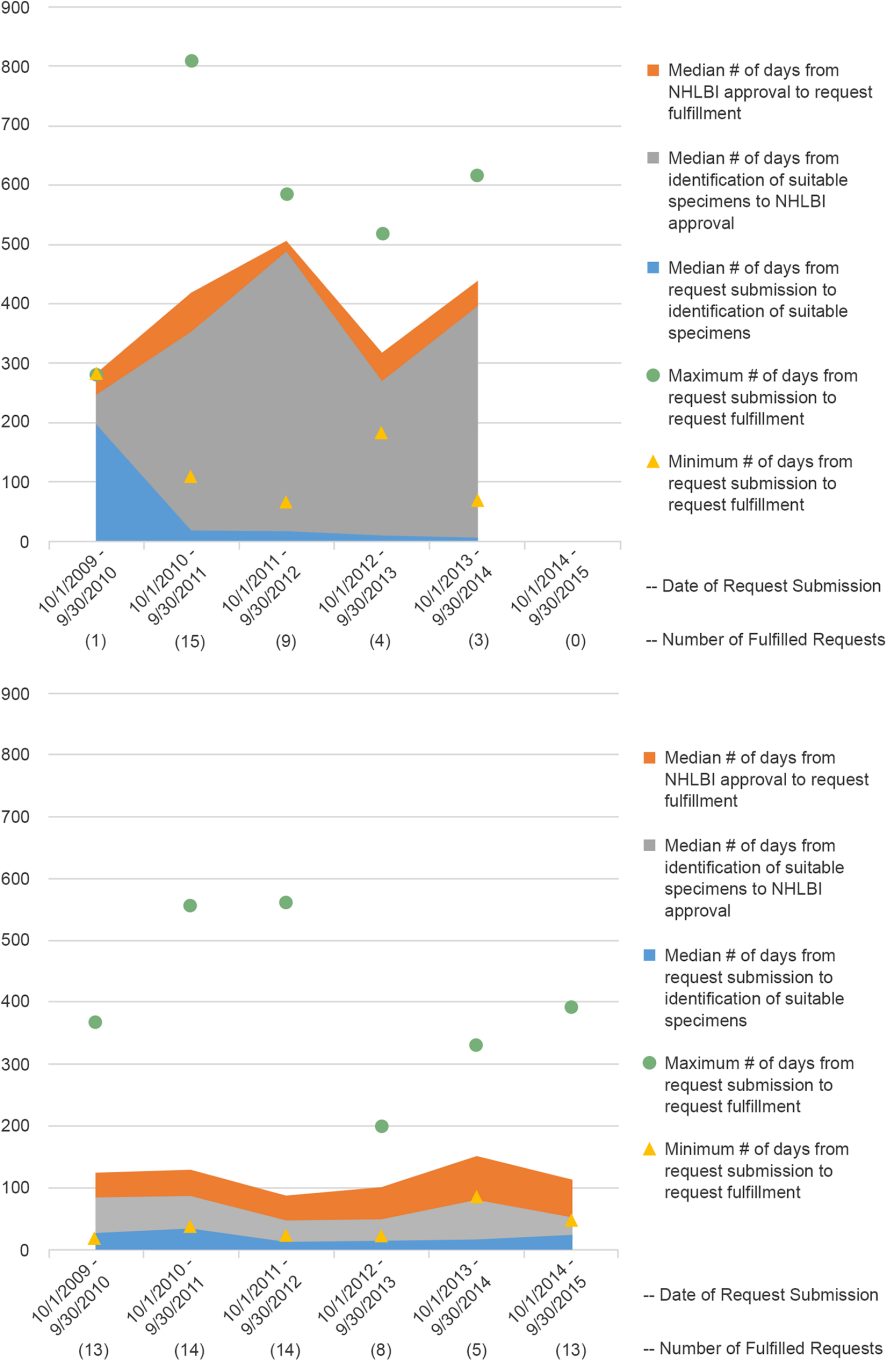
**Medians of time (in days) within biospecimen request processing sub-intervals for requests seeking funding (2a) and for requests with pre-existing funding (2b) by program year**.

The time to fulfillment is comprised of three sub-intervals which may be examined independently: the initial search for appropriate biospecimens; the wait time for requestor-supplied documentation such as IRB approvals and Material Transfer Agreement (MTA) signatures and funding actions; and the preparation of biospecimens for shipment. All biospecimen requests must demonstrate that funding is available for the assays to be performed. Requests for biospecimens which are submitted without available funding are put on hold until such funding is available, and this has a significant impact on time to fulfillment. Fig 2A and 2B are area stack graphs which plot the medians of each of the sub-intervals for requests which are seeking funding at the time of the submission (Fig 2A) vs. those submitted with pre-existing funding (Fig 2B). Time to fulfill requests with pre-existing funding fell from a median of 164 days in the first year to 117 days in the sixth year, remaining steady for the last four years. In contrast, the time to fulfill requests which are seeking funding has remained steady, ranging from a median of 425 days in the second year to 432 days in the sixth year (disregarding the first year where only one such request was fulfilled), and can be seen to be largely due to the wait for funding which has ranged from a high of 472 days in the third year to a low of 260 days in the fourth year. The time spent in the other two phases has shown a decrease, from 18 days to 6 days to search for appropriate biospecimens and from 66 days to 42 days to prepare the biospecimens for shipping, again disregarding the initial year.

**Fig 3:**
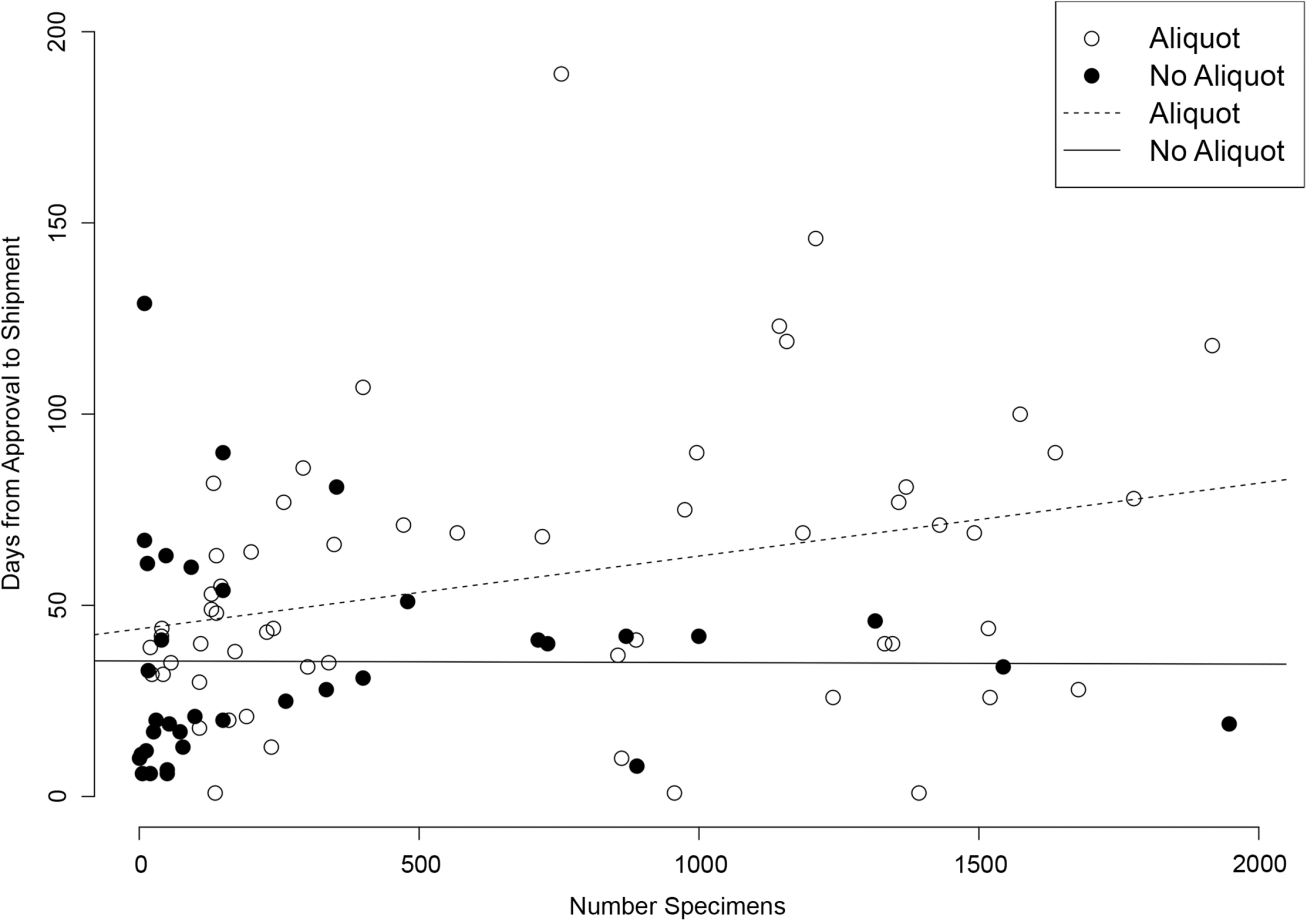
Time to fulfillment of biospecimen requests by aliquot requirement status.

The impact of aliquoting biospecimens and the number of requested biospecimens on the preparation and shipping time was investigated. Although BioLINCC does not offer custom aliquoting services to recipients, aliquoting may be performed as part of the request fulfillment process in order to preserve the collections. Fig 3 displays all requests for 2000 or fewer biospecimens with durations from submission to fulfillment of 0 to 200 days. The preparation time for biospecimens which required no aliquoting had times to fulfillment which were independent of the number of biospecimens requested, while times to fulfillment for requests that did require aliquoting increased with increasing numbers of biospecimens requested. Aliquoting is a significant investment in the management of the Biorepository resources and is only undertaken after careful consideration of collection utilization and scientific value.

### Requestor metrics

One of the primary goals of the BioLINCC program is to encourage the use of biospecimen collections among investigators early in their career and among investigators who were not part of the original research. Table 3 displays the number of years of experience of the primary investigator into 5-year categories (29% had less than 10 years of experience). It also demonstrates that most (87%) of the researchers who obtain specimens through BioLINCC were not affiliated with the original study).

**Table 3:** Requestor characteristics by website year.

	PI—Years of Research Experience			PI Involved in Original Research Study		Total
Website Year	0–5 Years	5–10 Years	10+ Years	Yes	No	
10/1/2009–9/30/2010	3	1	10	2	12	14
10/1/2010–9/30/2011	2	5	22	2	27	29
10/1/2011–9/30/2012	3	7	13	5	18	23
10/1/2012–9/30/2013	1	3	8	0	12	12
10/1/2013–9/30/2014	1	1	6	1	7	8
10/1/2014–9/30/2015	1	1	11	3	10	13
Total	11	18	70	13	86	99

Table 4 breaks down the funding source for the intended research. It illustrates that 19% of the fulfilled requests were funded by applications to the NHLBI funding opportunity RFA-HL-12-004, Maximizing the Scientific Value of the NHLBI Biologic Specimen Repository: Scientific Opportunities (R21),—in the second and third years of the program. An additional 56% were funded with other federal or state funds. Twenty-four percent of the fulfilled requests were funded with non-government funds.

**Table 4:** Number of biospecimen requests by funding source and website year.

	Funding Source								
Website Year	Institutional/ Departmental	Non-NIH Federal Funding	Targeted NHLBI R21 Grant	NIH Intramural	NIH Extramural	Funding Outside USA	State Funding	Private Foundation	Total
10/1/2009–9/30/2010	2	1	0	4	7	0	0	0	14
10/1/2010–9/30/2011	3	1	11	2	9	2	0	1	29
10/1/2011–9/30/2012	1	0	8	1	7	2	1	3	23
10/1/2012–9/30/2013	2	0	0	1	8	0	0	1	12
10/1/2013–9/30/2014	3	0	0	0	5	0	0	0	8
10/1/2014–9/30/2015	4	1	0	0	8	0	0	0	13
Total	15	3	19	8	44	4	1	5	99

To date, thirty-two successful requests have resulted in at least one published manuscript from the research performed on the biospecimens (Table 5). Additional publications are expected to result from requests initiated in the last 2–3 program years. Lag time components from request to publication include request searching and fulfillment, assay/research run time, and manuscript preparation/publication. The current publication count is 73.

**Table 5:** Publications by website request year.

	Publications		
Website Year	Number of Fulfilled Requests	Requests with at Least One Publication	Highest Journal Impact Factor
10/1/2009–9/30/2010	14	4	9
10/1/2010–9/30/2011	29	15	28
10/1/2011–9/30/2012	23	10	9
10/1/2012–9/30/2013	12	3	11
10/1/2013–9/30/2014	8	0	NA
10/1/2014–9/30/2015	13	0	NA
Total	99	32	28

### Biospecimen utilization

At the end of the sixth year of the BioLINCC program, 41 collections were available for biospecimen distribution: 19 sponsored by the NHLBI Division of Blood Diseases and Resources (DBDR), 11 by the Division of Lung Diseases (DLD), and 11 by the Division of Cardiovascular Sciences (DCVS), comprising nearly 3.8 million specimens [1,555,002 serum, 1,135,639 plasma, 80,446 DNA, 508,872 whole blood, 67,583 urine, 214,918 buffy coat, 36,728 red blood cells, 4,647 bronchoalveolar lavage (BAL), 108,706 peripheral blood mononuclear cells (PBMC), and 79,852 of other material types]. During the first six years, 118,702 biospecimens (30,484 serum, 43,062 plasma, 10,486 DNA, 19,125 whole blood, 8,938 urine, 2,151 buffy coat, 1,706 red blood cells, 644 BAL, 266 PBMC, and 1,840 of other material types) were distributed from 31 of those collections (7 DLD, 14 DBDR, 10 DCVS).

**Fig 4:**
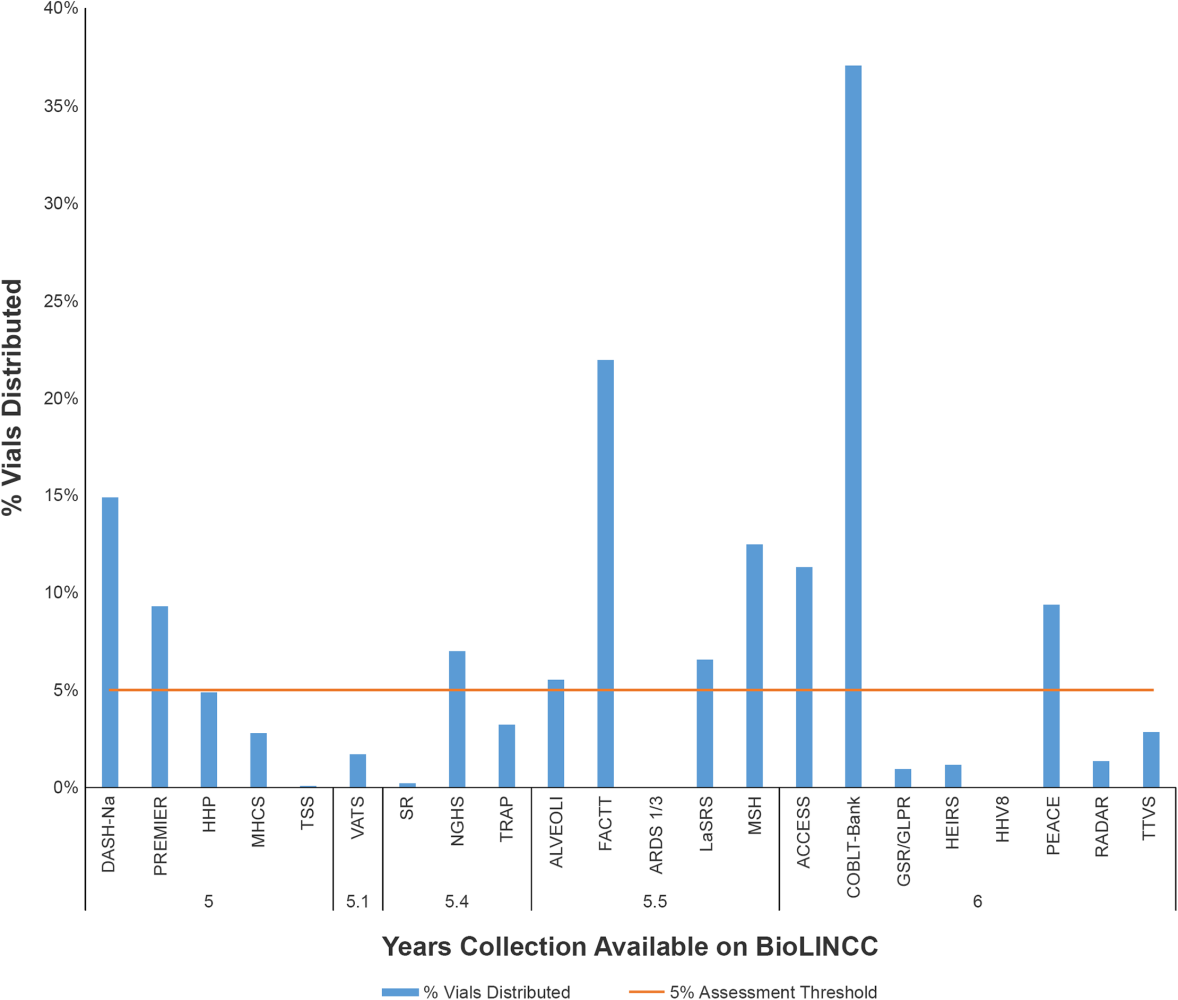
Utilization percentages for biospecimen collections that have been available on BioLINCC for 5 years or more.

The utilization of collections that have been available online through BioLINCC for at least 5 years is monitored by the NHLBI, and collections with a utilization percentage of less than 5 percent are reviewed to determine if the collection size should be reduced. Fig 4 shows the utilization percentages for 22 collections that have been available through BioLINCC for at least five years. The full study names for the acronyms used in this figure are provided in S1 Table, and links to full study descriptions on the BioLINCC website are included. Ten of these collections have exceeded the five percent target in the first six years, and five collections have had less than one percent utilization.

## Discussion

The NHLBI Biologic Specimen and Data Repositories represent a significant investment of resources both in terms of the funding and scientific oversight of the Parent Studies in the conduct of the original research and in the maintenance and storage costs associated with long-term storage of research resources. It is in the Institute’s interest to ensure that the program is meeting its goals and is responsive to the overall strategic plan. Program metrics can provide evidence of success or identify areas for improvement. To ensure optimal resource management, and to identify areas where resources may be under-utilized by the target research community, program metrics were designed at the outset and have been enhanced as the BioLINCC program and methods evolved over time.

Program visibility, as measured by website metrics, indicate a healthy activity rate and demonstrate that new users continue to come to the site. The BioLINCC resource has been promoted regularly since the website was established in October 2009. Program awareness activities have included posters, oral presentations and informational booths at well-attended scientific conferences several times annually, as well as the development and distribution of educational materials both within various NIH Institutes and at additional external conferences which focus on either biobanking science or on specific disease areas (e.g., American Heart Association, American Society of Hematology, American Thoracic Society, etc.). The program has also been promoted with NIH NHLBI R21 funding opportunities, including a two-year cycle made available beginning in its second year. Seventy-six requests were submitted for funding via the R21 mechanism in those cycles (19 of these were funded and fulfilled). More recently, a three-year cycle was made available with a start date in early 2017, (RFA-HL-17-022 http://grants.nih.gov/grants/guide/rfa-files/RFA-HL-17-022.html).

Request metrics were designed and developed to both provide the Institute with gauges to monitor the efficiency of internal processes and to identify areas where there was room for improvement in review and approval processes; data-driven determinants of the impact of requests on collections based upon high-interest or rare specimens vs. low-impact vials; and refinements to request fulfillment approaches at the Biorepository during the peak request periods which result when targeted grant funding is available. By separating and examining fulfillment time subintervals separately (rather than as a single overall time interval, it became clear that long intervals were generally the result of funding issues rather than internal program factors.

Requestor metrics were developed to provide information on the degree to which the program is able to reach early-stage investigators—who generally do not have the fiscal resources for large research efforts—to encourage innovative thought and hypothesis generation. Our data demonstrate that about 11% of our recipients have fewer than 5 years of research experience, with an additional 18% mid-stage, defined as between 5–10 years. A possible weakness in our metrics is our sense that although more senior investigators submit the request, the research itself is driven by more junior investigators, and we continue to explore how to get a more reliable indicator of this metric. We are more confident in our data regarding whether the requestor was involved in the original Parent Study, and are pleased to see that a majority of researchers who request biospecimens through the BioLINCC program are no longer from the original Parent Study groups. Sustainability of historic biospecimen archival collections is only possible if new users access the specimens.

Approximately one third (32/99) of the fulfilled requests had been identified as resulting in at least one publication by the September 2016 cutoff for inclusion in this manuscript. Unless notified by the recipient that the research project has been completed, we query on an annual basis for research status and to update the research publication lists. For researchers who performed their research under NIH grant funding mechanisms, the grantee progress reports are also monitored by NHLBI program staff and additional information on any publications is added to the BioLINCC records from that source. There are several possible factors in the lag time between specimen delivery to the researcher and publication, including the time shift in the actual work, its interpretation, and preparation/final acceptance of a manuscript. Anecdotally, we are also aware of several early exploratory projects which went directly to larger validation projects that are still in design or in process, and we are hopeful that these downstream results will be published. We are pleased that our publication rate thus far has been as positive as it has been, given that a third of NIH-funded Phase II or later clinical trials were found to have remained unpublished by a median of 4.25 years after study completion [3]. That being said, all requests fulfilled through the BioLINCC program required documentation of scientific review of the merit of the hypothesis and the proposed technical approach. Therefore, any research which resulted in negative findings would still contribute to researchers’ scientific knowledge and could result in a new question or hypothesis. However, publications are both labor-intensive and expensive to prepare and publish. An inexpensive and straight-forward mechanism for researchers to share negative-outcome projects (hypothesis/methods/findings), for example through a PubMed or a separate US National Library of Medicine database, would be a useful addition to the scientific literature.

The metrics have also been incorporated into the twice-yearly evaluation by the NHLBI of each collection’s utility as a scientific resource. The evaluation examines the uniqueness of the collection, the extent and completeness of associated data and documentation, the number of requests submitted, the number of requests fulfilled and the projected maintenance cost. Collections with less than 5% use (the number of biospecimens distributed from the collection when posted on BioLINCC) after five years of online access are selected for review and reports describing past use and the cost of reductions versus maintenance are prepared. The reports are then used to develop strategies to promote use and/or reduce the number of vials stored, with input from the original study investigators when available. In addition, the data-to-inventory links established to search for biospecimens lead to the development of an informatics freezer visualization tool that has dramatically improved the management of freezer space by reducing the cost of removing vials with no/low utility and consolidating vials to reduce the number of storage boxes. In two years, 23 collections (over 1.2 million vials) were consolidated, freeing up space equivalent to 10 freezers and over one million vials of no/low utility were removed from the inventory [4]. This reduced the maintenance cost of the Biorepository by an estimated 25% a year.

## Conclusions

The metrics used to monitor the BioLINCC program activities have proven to be invaluable. They have improved Biorepository workflows and provide both insight into the scientific utility of a collection and into the use and users of the biospecimens that otherwise would not be possible. Of particular interest to us were the data we collected on the characterization of the type of proposed research. Scientific impact is often tied to publication, but the BioLINCC data demonstrate that basing impact on publications alone without understanding the landscape of the research being performed may be misleading. Being able to access biospecimens to test a hypothesis may not result in a manuscript but does result in addressing a scientific question.

## Supporting information

S1 Table Study names and references corresponding to the study acronyms used in Fig 4. (XLSX)

S2 Table Data referenced in this paper. (XLSX)

S3 Table Mapping of supplemental data to tables and figures. (DOCX)
