# Supplementary material for: Providing researchers with online access to NHLBI biospecimen collections: The results of the first six years of the NHLBI BioLINCC program
Source: PLoS One. 2017 Jun 14;12(6):e0178141. doi: 10.1371/journal.pone.0178141 (PMC5470669; doi:10.1371/journal.pone.0178141)
Supplement: S3 Table — (DOCX) [file pone.0178141.s003.docx]

**Figure 1**

Source Table: Data - Website User Metrics

Filters: None

| **Figure Element** | **Source Data Variable(s)** | **Data Manipulations/Calculations** |
| --- | --- | --- |
| Website Year | Website Year (Column A) | None |
| New Unique IPs | New Unique IPs (Column C) | None |
| Cumulative Unique IPs | Cumulative Unique IPs (Column D) | None |

**Figure 2a**

Source Table: Data - Years 1 - 6 Requests

Filters: Request Status (Column C) = “Fulfilled”; Funding Available at Time of Request (Column AR) = “No”

| **Figure Element** | **Source Data Variable(s)** | **Data Manipulations/Calculations** |
| --- | --- | --- |
| Date of Request Submission | Online Year (Column D) | Year 1 maps to 10/1/2009 – 9/30/2010  Year 2 maps to 10/1/2010 – 9/30/2011  Year 3 maps to 10/1/2011 – 9/30/2012  Year 4 maps to 10/1/2012 – 9/30/2013  Year 5 maps to 10/1/2013 – 9/30/2014  Year 6 maps to 10/1/2014 – 9/30/2015 |
| Number of Fulfilled Requests | Online Year (Column D) | Count (N) by Online Year |
| Median # of days from NHLBI approval to request fulfillment | Time (days): Date Approved to Date Specimens Shipped (Column M) | Median by Online Year |
| Median # of days from identification of suitable specimens to NHLBI approval | Time (days): Date of Successful Search to Date of Approval (Column L) | Median by Online Year |
| Median # of days from request submission to identification of suitable specimens | Time (days): Date Created to Date of Successful Search (Column K) | Median by Online Year |
| Maximum # of days from request submission to request fulfillment | Time (days): Date Created to Date Specimens Shipped (Column J) | Maximum by Online Year |
| Minimum # of days from request submission to request fulfillment | Time (days): Date Created to Date Specimens Shipped (Column J) | Minimum by Online Year |

**Figure 2b**

Source Table: Data - Years 1 - 6 Requests

Filters: Request Status (Column C) = “Fulfilled”; Funding Available at Time of Request (Column AR) = “Yes”

| **Figure Element** | **Source Data Variable(s)** | **Data Manipulations/Calculations** |
| --- | --- | --- |
| Date of Request Submission | Online Year (Column D) | Year 1 maps to 10/1/2009 – 9/30/2010  Year 2 maps to 10/1/2010 – 9/30/2011  Year 3 maps to 10/1/2011 – 9/30/2012  Year 4 maps to 10/1/2012 – 9/30/2013  Year 5 maps to 10/1/2013 – 9/30/2014  Year 6 maps to 10/1/2014 – 9/30/2015 |
| Number of Fulfilled Requests | Online Year (Column D) | Count (N) by Online Year |
| Median # of days from NHLBI approval to request fulfillment | Time (days): Date Approved to Date Specimens Shipped (Column M) | Median by Online Year |
| Median # of days from identification of suitable specimens to NHLBI approval | Time (days): Date of Successful Search to Date of Approval (Column L) | Median by Online Year |
| Median # of days from request submission to identification of suitable specimens | Time (days): Date Created to Date of Successful Search (Column K) | Median by Online Year |
| Maximum # of days from request submission to request fulfillment | Time (days): Date Created to Date Specimens Shipped (Column J) | Maximum by Online Year |
| Minimum # of days from request submission to request fulfillment | Time (days): Date Created to Date Specimens Shipped (Column J) | Minimum by Online Year |

**Figure 3**

Source Table: Data - Years 1 - 6 Requests

Filters: Request Status (Column C) = “Fulfilled”; Specimen # (Column O) < 2000; Time (days): Date Approved to Date Specimens Shipped < 200

| **Figure Element** | **Source Data Variable(s)** | **Data Manipulations/Calculations** |
| --- | --- | --- |
| Number of Specimens | Specimen # (Column O) | None |
| Days from Approval to Shipment | Time (days): Date Approved to Date Specimens Shipped (Column M) | None |
| Aliquot (Data Point) | Any Specimens Aliquoted? (Column N) | Exclude “No” values |
| No Aliquot (Data Point) | Any Specimens Aliquoted? (Column N) | Exclude “Yes” values |

The plot was generated in R with the plot() function. The aliquot and no aliquot lines of best fit were generated using the abline() function.

**Figure 4**

Source Table: Data - Study Utilization

Filters: # of Years Open on BioLINCC (Column D) ≥ 5

| **Figure Element** | **Source Data Variable(s)** | **Data Manipulations/Calculations** |
| --- | --- | --- |
| Years Collection Available on BioLINCC | Study (Column A) and # of Years Open on BioLINCC (Column D) | None |
| % Vials Distributed | Usage Since Website Launch (Column E) | None |

**Table 1**

Source Table: Data - Years 1 - 6 Requests

Filters: None

| **Table Element** | **Source Data Variable(s)** | **Data Manipulations/Calculations** |
| --- | --- | --- |
| Website Year | Online Year (Column D) | Year 1 maps to 10/1/2009 – 9/30/2010  Year 2 maps to 10/1/2010 – 9/30/2011  Year 3 maps to 10/1/2011 – 9/30/2012  Year 4 maps to 10/1/2012 – 9/30/2013  Year 5 maps to 10/1/2013 – 9/30/2014  Year 6 maps to 10/1/2014 – 9/30/2015 |
| Request Disposition | Request Status (Column C) | Closed - request not funded, Closed – inactivity, Closed - cancelled by user - other reason / NOS, Closed - request requirements, Closed - request not funded, and Closed - denied map to Unsuccessful |
| Research Type (Fulfilled Requests Only) | Research Type (Column AV) | Include only requests with Request Status = Fulfilled |

**Table 2**

Source Table: Data - Years 1 - 6 Requests

Filters: Request Status (Column C) ≠ “Fulfilled”

| **Table Element** | **Source Data Variable(s)** | **Data Manipulations/Calculations** |
| --- | --- | --- |
| Website Year | Online Year (Column D) | Year 1 maps to 10/1/2009 – 9/30/2010  Year 2 maps to 10/1/2010 – 9/30/2011  Year 3 maps to 10/1/2011 – 9/30/2012  Year 4 maps to 10/1/2012 – 9/30/2013  Year 5 maps to 10/1/2013 – 9/30/2014  Year 6 maps to 10/1/2014 – 9/30/2015 |
| Reason Not Fulfilled | Request Status (Column C) | Closed - request not funded maps to Request Not Funded  Closed - inactivity maps to Inactivity  Closed - cancelled by user - other reason / NOS maps to Cancelled by User/NOS  Closed - request requirements maps to Request Requirements  Closed - request not funded maps to Request Not Funded  Closed - denied maps to Denied |

**Table 3**

Source Table: Data - Years 1 - 6 Requests

Filters: Request Status (Column C) = “Fulfilled”

| **Table Element** | **Source Data Variable(s)** | **Data Manipulations/Calculations** |
| --- | --- | --- |
| Website Year | Online Year (Column D) | Year 1 maps to 10/1/2009 – 9/30/2010  Year 2 maps to 10/1/2010 – 9/30/2011  Year 3 maps to 10/1/2011 – 9/30/2012  Year 4 maps to 10/1/2012 – 9/30/2013  Year 5 maps to 10/1/2013 – 9/30/2014  Year 6 maps to 10/1/2014 – 9/30/2015 |
| PI - Years of Research Experience | PI Status (Years In Scientific Research) (Column AT) | None |
| PI Involved in Original Research Study | Original Study PI? (Column AP) | None |

**Table 4**

Source Table: Data - Years 1 - 6 Requests

Filters: Request Status (Column C) = “Fulfilled”

| **Table Element** | **Source Data Variable(s)** | **Data Manipulations/Calculations** |
| --- | --- | --- |
| Website Year | Online Year (Column D) | Year 1 maps to 10/1/2009 – 9/30/2010  Year 2 maps to 10/1/2010 – 9/30/2011  Year 3 maps to 10/1/2011 – 9/30/2012  Year 4 maps to 10/1/2012 – 9/30/2013  Year 5 maps to 10/1/2013 – 9/30/2014  Year 6 maps to 10/1/2014 – 9/30/2015 |
| Funding Source | Funding Source (Column AS) | NIH Extramural + Type of award: R, T, K, U or Other maps to NIH Extramural  Funding Outside of the United States maps to Funding Outside USA  NIH Intramural Research maps to NIH Intramural  RFA-HL-12-004 maps to Targeted NHLBI R21 Grant |

**Table 5**

Source Table: Data - Years 1 - 6 Requests

Filters: Request Status (Column C) = “Fulfilled”

| **Table Element** | **Source Data Variable(s)** | **Data Manipulations/Calculations** |
| --- | --- | --- |
| Website Year | Online Year (Column D) | Year 1 maps to 10/1/2009 – 9/30/2010  Year 2 maps to 10/1/2010 – 9/30/2011  Year 3 maps to 10/1/2011 – 9/30/2012  Year 4 maps to 10/1/2012 – 9/30/2013  Year 5 maps to 10/1/2013 – 9/30/2014  Year 6 maps to 10/1/2014 – 9/30/2015 |
| Number of Fulfilled Requests | Online Year (Column D) | Count (N) by Online Year |
| Requests with at Least One Publication | Publication Count (Column AW) | Count (N) by Online Year of requests with ≥ 1 publication |
| Highest Journal Impact Factor | Highest Journal Impact Factor (Column AX) | Maximum by Online Year |
